# Consensus Copy-Number Alteration Signatures from Clinical Panels Enable Pan-Cancer Risk Stratification and Therapy Response Association

**DOI:** 10.3390/ijms27041764

**Published:** 2026-02-12

**Authors:** Adar Yaacov

**Affiliations:** 1 Helmsley Cancer Center, Shaare Zedek Medical Center, Jerusalem 9103102, Israel; adar.yaacov@mail.huji.ac.il; 2 Faculty of Medicine, The Hebrew University of Jerusalem, Jerusalem 7610001, Israel

**Keywords:** cancer genomics, copy-number alteration signatures, precision oncology, biomarkers, deconvolution algorithms

## Abstract

Somatic copy-number alterations (CNAs) are pervasive in cancer, but routine targeted panels yield sparse CNA readouts unsuited for CNA signature analysis. We built a consensus framework that integrates four deconvolution algorithms to extract CNA signatures from panel data. Analysis of 24,870 tumors sequenced using MSK-IMPACT identified five reproducible signatures (CON1–CON5). CON5 mirrored near-diploid profiles, whereas the others captured distinct aneuploid patterns. Technical fidelity was confirmed by internal cross-validation and external validation in sarcoma and hepatocellular carcinoma cohorts. Clinically, these signatures were associated with overall survival across tumor types (hazard ratio 1.3–2.5; FDR < 0.01) and provided additive prognostic information beyond Fraction of Genome Altered. Associations with driver mutations (GATA3 in CON1, KRAS in CON5) supported biological specificity, and the signatures delineated resistance landscapes for chemotherapy, hormonal, targeted, and immunotherapy. By converting routine panel data into biologically interpretable prognostic features, our framework enables risk stratification and therapeutic guidance in precision oncology.

## 1. Introduction

Somatic copy-number alterations (CNAs) are common events in cancer, reshaping the cancer genome by deleting tumor suppressors, amplifying oncogenes, and remodeling chromosomal architecture [1,2,3]. Pan-cancer surveys have shown that >90% of tumors harbor recurrent CNAs, often encompassing tens to hundreds of megabases [1]. Beyond individual lesions, aggregated CNA patterns, so-called “CNA signatures”, capture the history of genome instability and can stratify prognosis, infer DNA repair defects, and nominate therapeutic vulnerabilities [4,5]. However, the discovery of these signatures has been dependent on high-resolution platforms like whole-genome sequencing (WGS) or SNP arrays [4,5]. While powerful, these technologies remain largely confined to research settings due to cost and logistical hurdles, precluding their use in routine oncology practice, where targeted next-generation sequencing (NGS) panels predominate [6,7,8,9]. These targeted panels, such as those by MSK-IMPACT and FoundationOne, simultaneously profile hundreds of actionable cancer genes in tens of thousands of patients annually [10,11,12]. They permit approximate copy-number calling, yet the resultant data are sparse, covering <2% of the genome, and noisy, limiting direct transfer of WGS-derived signature methods [13]. Consequently, mutational signatures have not been incorporated into most clinical genomic reports, representing an untapped reservoir of prognostic and predictive information. Recent studies developed computational tools that detect single nucleotide variant (SNV) mutational signatures from targeted gene panels [14,15]. However, no such method exists for copy-number signatures.

The challenge is compounded by methodological limitations. Current approaches for signature discovery typically rely on a single computational algorithm, such as non-negative matrix factorization (NMF) and hierarchical Dirichlet processes (HDP), each of which captures different aspects of the underlying biology but suffers from algorithm-specific biases [16,17]. To address these challenges, we reasoned that a consensus-based approach, which integrates the outputs of multiple complementary algorithms, could extract robust CNA signatures even from the sparse data inherent to targeted panel sequencing. Such ensemble frameworks have demonstrated superior performance and generalizability in other areas of bioinformatics by mitigating method-specific biases and capturing a more comprehensive biological signal [18]. However, no such consensus framework has been developed specifically for CNA signature analysis, representing a critical gap in the computational toolkit for cancer genomics.

Here, we present a consensus-driven computational pipeline that combines Independent Component Analysis (ICA), NMF, HDP, and Graph-based NMF Deconvolution (GD) to infer recurring CNA signatures from targeted panel data. Applying this approach to MSK-IMPACT profiles of 24,870 tumors across diverse histologies from the MSK-CHORD cohort [19], we uncovered five reproducible signatures (CON1–5) with distinct genomic topographies and prognostic associations. We demonstrate that these signatures have distinct genomic features, are associated with clinical outcomes, and show superior prognostic performance compared to single-algorithm approaches.

## 2. Results

### 2.1. Copy-Number Signatures from Targeted Sequencing Panels

**Figure 1:**
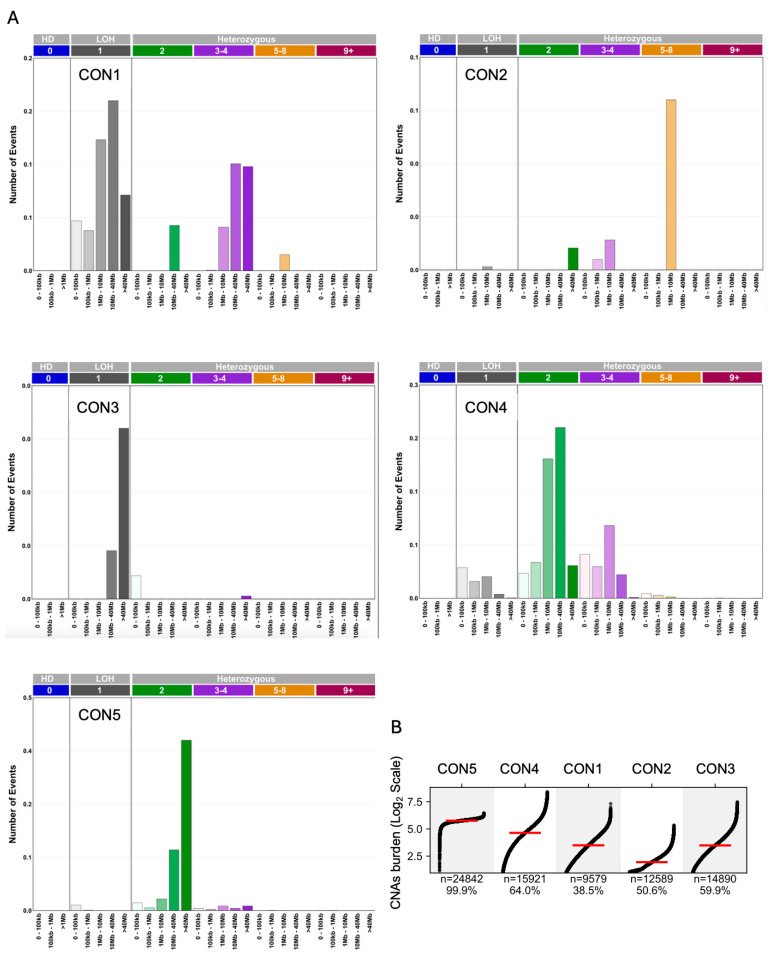
Discovery and characterization of five consensus copy-number signatures. (**A**) Bar plots defining the composition of each signature. The y-axis shows the relative contribution of 28 CNA features, categorized by copy-number state (HD, homozygous deletion; LOH, loss of heterozygosity; gains from 3 to 4 to 9+ copies) and CNA segment size (from <100 Kb to >40 Mb). (**B**) Scatter plot illustrating the contribution of each of the five consensus signatures (CON1–5) to the total copy-number alteration (CNA) burden (Log2 scale) in over 24,000 tumors from the discovery cohort. Each point represents a single tumor. The prevalence of each signature and the number of tumors with a non-zero contribution (*n*) are noted. Red line marks the median contribution of the given signature.

To identify robust copy-number alteration (CNA) signatures from sparse clinical sequencing data, a multi-algorithm “consensus” framework was developed (see Section 4 and Figure 1). The framework was applied to a 5-cancer-type cohort of 24,870 tumors from patients who underwent sequencing with the MSK-IMPACT targeted gene panel [19]. For each tumor, CNA events were systematically called and classified into 28 distinct features based on CNA type (homozygous deletion, loss of heterozygosity (LOH), and amplification) and the length of the genomic region affected. The publicly available data lack LOH annotation, so LOH regions with more than one copy are not included in the classification system (See Section 4). This high-dimensional feature matrix served as the input for four independent, complementary pattern deconvolution algorithms: ICA, NMF, HDP, and GD. The application of these methods initially yielded a set of 4–15 primary signatures across the four approaches. This variability across different methods necessitated a consensus-driven approach to delineate robust signatures.

### 2.2. A Consensus-Driven Approach Yields Five CNA Signatures

To distill a stable set of signatures independent of the biases of any single algorithm, a consensus clustering strategy was implemented. First, pairwise cosine similarity between all primary signatures extracted by the four methods was computed, revealing clusters of highly related signatures derived from different algorithms (Supplementary Figure S1). Then, using hierarchical clustering on this similarity matrix, we identified five distinct and robust signature clusters (Section 4). The primary signatures within each cluster were merged to form five final consensus signatures, designated CON1 through CON5 (Figure 1). This approach ensures that each consensus signature represents a pattern detected by a diversity of computational methods, increasing confidence in its biological relevance.

Each of the five consensus signatures was defined by a unique combination of CNA features (Figure 1A). CON5 was characterized almost exclusively by large heterozygous genomic segments, reflecting a diploid or near-diploid genome state. CON4 was a mixed signature, composed of multiple CNA categories of various lengths, including LOH, heterozygous segments, and low-level amplifications (3–4 copies). In contrast, CON3 was strongly enriched for large LOH segments. CON1 also featured LOH and 3–4 copy amplifications, but was distinguished by a strong prevalence of large-scale events affecting substantial portions of chromosomal arms. Finally, CON2 represented a rarer genomic phenomenon, characterized by focal amplifications (1–10 Mb) with intermediate copy-number gains (5–8 copies). The five signatures exhibited widely different prevalence and activity levels across the 24,870 tumors (Figure 1B). As expected for a signature reflecting a diploid state, CON5 was active in 99.9% of samples and displayed the highest median burden (activity score of ~6.0, log2 scale). CON4 was the next most common, found in 64% of tumors with a high median burden of ~4.5. CON3 was also prevalent, identified in 59.9% of samples, with a median burden of ~3.5. CON1 was present in 38.5% of tumors, with a similar median burden to CON3 (~3.5). While CON2 was detected in 50% of samples, its contribution to the overall CNA landscape in most of these cases was very small, indicating it may often represent a minor subclonal event. Homozygous deletions (HDs) and high-level amplifications (9+ copies) were absent across all signatures, reflecting the rarity of these CNA events in the published targeted NGS data, and consistent with COSMIC WGS-derived signatures, which similarly show low contributions from these categories.

Comparing the targeted panel-based CON1–5 to the COSMIC CN signatures derived from WGS/SNP by cosine similarity, CON5 and CN1 were almost identical (cosine similarity of 0.98), while the other CON signatures had lower but still relatively high similarity with the COSMIC CN signatures, ranging 0.72–0.84 to signatures with etiologies like chromothripsis, chromosomal LOH (1 copy), focal LOH (1 copy) with genome duplication, and octoploidy (Supplementary Figure S2).

### 2.3. Prognostic Impact of CNA Signatures Across Cancer Types

**Figure 2:**
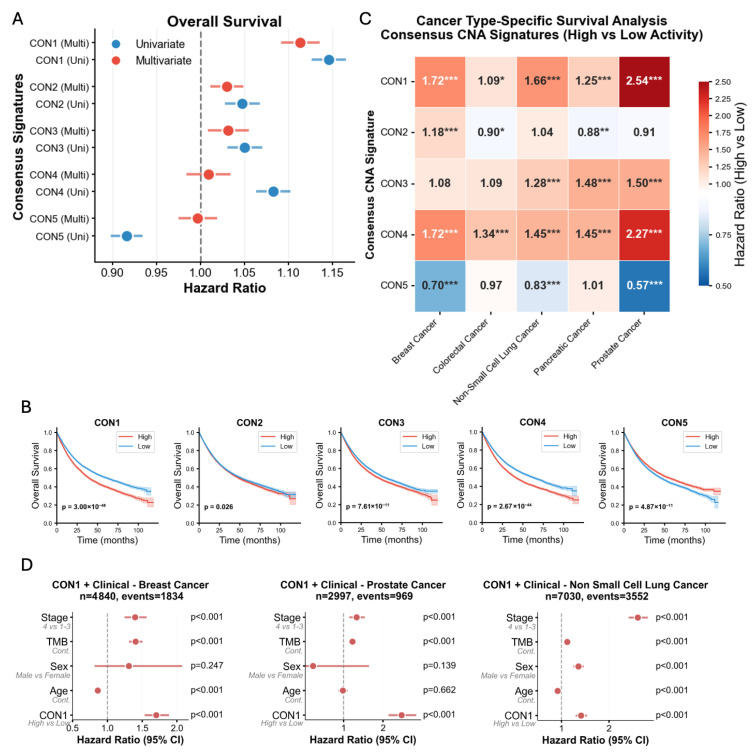
Prognostic impact of consensus CNA signatures on overall survival. (**A**) Forest plot of hazard ratios (HRs) from univariate and multivariable Cox proportional hazards models for each signature’s activity across the entire cohort. Multivariable models were adjusted for all signatures combined. Error bars represent 95% confidence intervals. (**B**) Pan-cancer Kaplan–Meier curves for overall survival, with patients stratified by high versus low activity (median split) for each of the five signatures. *p*-values were calculated using the log-rank test. The colored shading represents the 95% confidence intervals. (**C**) Heatmap displaying the cancer type-specific prognostic performance of each signature. Colors represent HR. Significance levels are denoted by asterisks (* *p* < 0.05, ** *p* < 0.01, *** *p* < 0.001). (**D**) Forest plots of multivariable OS analyses for CON1 signature along with stage, tumor mutational burden (TMB), age and sex, for breast (**left**), prostate (**middle**) and non-small cell lung cancer (**right**).

We next investigated the association between the activity of each signature and patient overall survival (OS) across the entire cohort. In a univariate Cox proportional hazards model, i.e., separately for each signature, with its exposure as a continuous variable, all signatures were associated with OS: CON1, CON2, CON3 and CON4 with worse OS and CON5 with better OS (Figure 2A). When grouping patients into a high/low classification of a given signature based on the median exposure value, Kaplan–Meier analyses confirmed that patients with high burdens of CON1, CON2, CON3, and CON4 had markedly poorer survival outcomes compared to patients with low burdens of these signatures, and an inverse relationship was observed with CON5 (Figure 2B). In a multivariable analysis, including all five signatures as covariates, CON1, CON2, and CON3 were each independently associated with significantly shorter OS (Figure 2A).

To determine if these prognostic associations were driven by specific malignancies, we performed cancer type-specific survival analyses. The negative prognostic impact of the key signatures was largely preserved across multiple major cancer types. High activity of CON1 was significantly associated with worse OS in all cancer types tested: prostate adenocarcinoma, breast cancer, colorectal cancer (CRC), pancreatic cancer and non-small cell lung cancer (NSCLC), with hazard ratios (HRs) ranging from 2.54 in prostate cancer (CI: 2.24–2.88, *p* = 1.3 × 10^−48^) to 1.72 in breast cancer (CI: 1.57–1.89, *p* = 7.9 × 10^−31^), 1.66 in NSCLC (CI: 1.55–1.76, *p* = 1.5 × 10^−55^), 1.25 in pancreatic cancer (CI: 1.14–1.38, *p* = 2.9 × 10^−6^) and 1.09 in CRC (CI: 1.02–1.19, *p* = 0.04) (Figure 2C). CON4 was also associated with worse OS across all cancer types: prostate (HR: 2.27, CI: 1.97–2.52, *p* = 5.4 × 10^−28^), breast (HR: 1.72, CI: 1.56–1.91, *p* = 2.2 × 10^−26^), pancreatic (HR: 1.45, CI: 1.33–1.58, *p* = 2.6 × 10^−17^), NSCLC (HR: 1.44, CI: 1.36–1.54, *p* = 4.4 × 10^−31^), and CRC (HR: 1.34, CI: 1.23–1.46, *p* = 7.8 × 10^−12^). Similarly, CON3 was statistically significantly associated with OS in NSCLC, pancreatic cancer and CRC (HRs: 1.28–1.50, *p* < 0.05). CON2 demonstrated mixed association: worse OS in breast cancer, slightly better OS in CRC and pancreatic cancer (HRs: 1.18, 0.90 and 0.88 respectively; all *p* < 0.05). Furthermore, multivariable OS analysis integrating sex, age, stage, and tumor mutational burden (TMB) confirmed independent OS association (Figure 2D). This demonstrates that the prognostic information captured by these CNA signatures is not confined to a single disease type, highlighting their broad clinical relevance.

**Figure 3:**
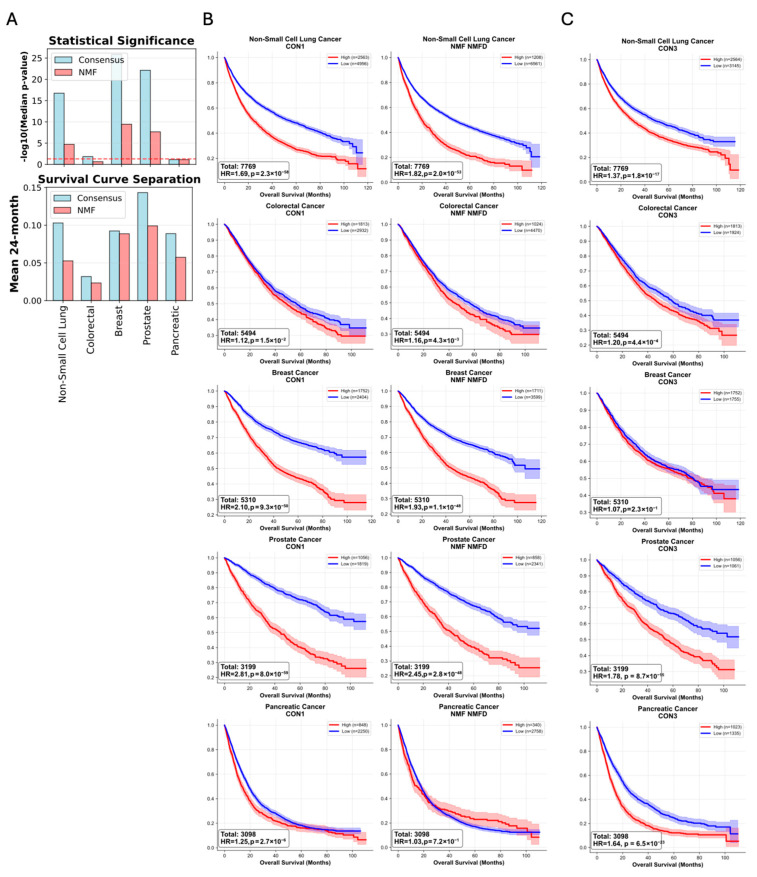
Superior prognostic performance of the consensus framework compared to a single NMF-based approach. (**A**) Summary bar charts directly comparing the performance of the consensus framework (blue) against the NMF-only method (red). Performance is measured by statistical significance (−log10 median *p*-value from log-rank tests) and the magnitude of survival curve separation (mean difference in survival probability at 24 months). The red dashed line represents *p*-value of 0.05. (**B**,**C**) Kaplan–Meier analyses of overall survival in all five cancer types (non-small cell lung, colorectal, breast, pancreatic and prostate cancers). Patients were stratified by high/low activity of consensus signatures (CON1 and CON3 shown as representative examples) versus signatures derived from a standard non-negative matrix factorization (NMF) approach. The colored shading represents the 95% confidence intervals.

To evaluate whether the consensus signatures offer an advantage over using a single method, we conducted a systematic comparison between the consensus and NMF signatures. The results demonstrated unanimous superiority of the consensus approach in their prognostic associations, both in terms of median *p*-value and mean 24-month survival (Figure 3A). Moreover, the differences are notable even in closely resembling signatures such as CON1 and NMFD. These are characterized by various lengths of LOH events, concurrently with a gain of 3–4 copies in large segments (Figure 1A and Supplementary Figure S3). However, CON1 provided a much stronger stratification of prognosis across all cancer types (Figure 3B). Meanwhile, CON3 signature, which is characterized by large (>40M bases) LOH events, has no equivalent in the NMF signatures, but is a strong prognostic marker across almost all cancer types, including with an HR > 1.5 in pancreatic cancer (*p* = 6.5 × 10^−23^) (Figure 3C). Beyond clinical superiority, consensus signatures demonstrated enhanced computational properties, including 16.9% lower inter-signature correlations (0.313 vs. 0.376), indicating better independence, and 2.3-fold higher information content (total variance 1662 vs. 737). These results provide compelling evidence that consensus signatures offer superior prognostic capability across diverse cancer contexts compared to traditional single-algorithm approaches.

### 2.4. Fitting CON Signatures in Tumors CNA Profiles

**Figure 4:**
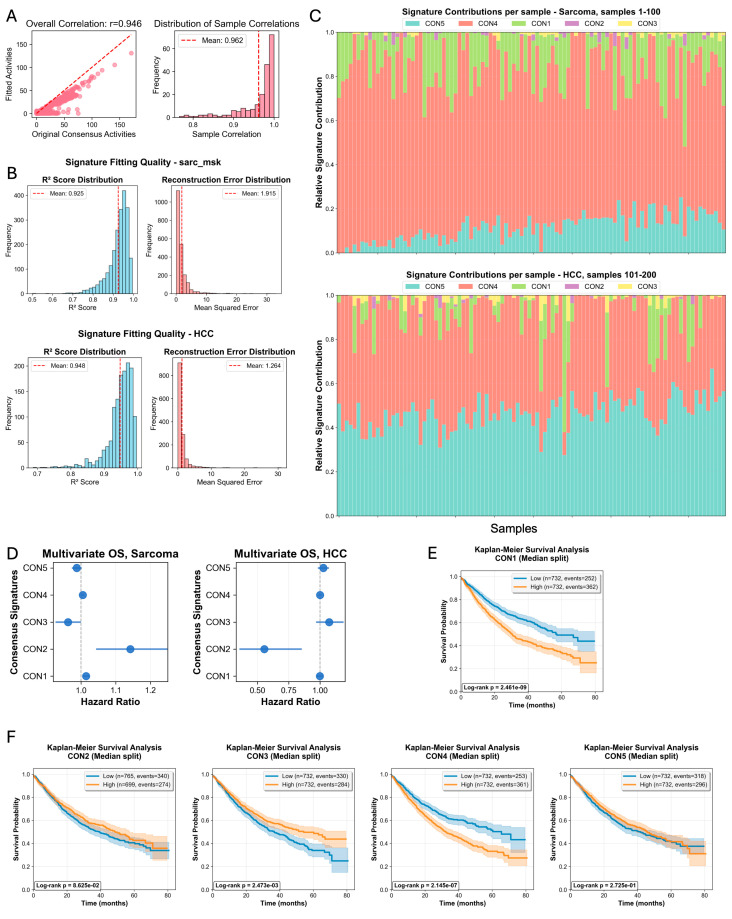
Development and independent validation of a signature fitting model. (**A**) Technical validation of the signature fitting method in 200 randomly selected samples, showing correlation between original extracted activities and fitted activities with the fitting approach. (**B**) Technical validation of the signature fitting method in two independent cohorts: sarcoma (**upper**) and hepatocellular carcinoma (HCC) (**lower**). Distributions of sample-wise R-squared scores (**left**), and mean squared reconstruction error (**right**) demonstrate high fitting fidelity. (**C**) Stacked bar plots showing signature contribution per sample in the independent sarcoma (**upper**) and HCC (**lower**) cohorts. Each bar represents a sample. (**D**) Clinical validation of the prognostic significance of the fitted signatures in the independent cohorts. Forest plots show multivariable hazard ratios for overall survival in sarcoma and HCC. (**E**,**F**) Kaplan–Meier curves show overall survival in the sarcoma validation cohort, stratified by fitted signature activity (high/low). The colored shading represents the 95% confidence intervals.

Next, we developed a fitting scheme to detect CNA signatures in new samples (see Section 4). In an internal validation set, we used 200 randomly selected samples from the original consensus signature training dataset to assess the accuracy of our fitting framework. The internal validation demonstrated exceptional performance across multiple metrics: The overall Pearson correlation between fitted and original signature activities reached 0.946 (95% CI: 0.935–0.957), with a corresponding coefficient of determination (R^2^) of 0.932, indicating that our fitting algorithm captured 93.2% of the variance in the original signature activities (Figure 4A). The reconstruction error was minimal (root mean squared error (RMSE) = 1.709), confirming high fidelity in signature activity estimation. Notably, 98% of samples (196/200) achieved high-quality fits (correlation > 0.8), with sample-level correlations ranging from 0.780 to 1.000 (mean = 0.962 ± 0.048) (Figure 4A). The non-negative least squares (NNLS) optimization method proved optimal, outperforming elastic net regression (R^2^ = 0.803). These internal validation results confirmed the reliability of our fitting framework before proceeding to independent external validation across diverse cancer types. To facilitate application, we developed an interactive web tool (https://consig.streamlit.app) that enables researchers to upload CNA data and obtain signature activities without requiring local software installation.

### 2.5. Independent Clinical Validation Reveals Cancer Type-Specific Prognostic Signatures

To test the robustness and generalizability of our findings, we applied the five established consensus signatures to independent validation cohorts of patients who were not part of the discovery cohort (Figure 4B). The activity of each signature was calculated (Figure 4C), and their prognostic value was assessed. Clinical validation in independent cohorts demonstrated significant prognostic associations for multiple CNA signatures with cancer type-specific patterns across two independent cohorts: MSK-Sarcoma and MSK-HCC (hepatocellular carcinoma). These datasets are independent in two aspects: new patients and new cancer types. In the sarcoma cohort (*n* = 1464, 614 events, median follow-up 21.8 months), four of five signatures showed significant associations with overall survival: CON1 (*p* < 0.001) and CON4 (*p* < 0.001) conferred increased mortality risk, while CON3 (*p* = 0.034) and CON5 (*p* = 0.046) were associated with improved survival. Kaplan–Meier analysis revealed pronounced survival differences, with CON4 and CON1 showing highly significant log-rank tests (*p* < 0.001 for both), where high-activity groups had substantially shorter median survival (32.4 vs. 65.5 months for CON4; 30.5 vs. 56.6 months for CON1) (Figure 4E,F). In contrast, the HCC cohort (*n* = 288, 164 events, median follow-up 24 months) showed a more focused pattern of associations. Importantly, CON2 was associated with improved outcomes (HR = 0.55, CI 0.36–0.85, *p* = 0.006). This pattern was already seen in colorectal and pancreatic cancers (Figure 2C), demonstrating the variability of the effect of high-amplitude amplifications of medium size—the hallmark of CON2. This validation in a distinct, homogenous cancer type confirms that our framework identifies fundamental biological patterns with durable prognostic power.

### 2.6. Consensus CNA Signatures Provide Prognostic Information Beyond Fraction of Genome Altered

**Figure 5:**
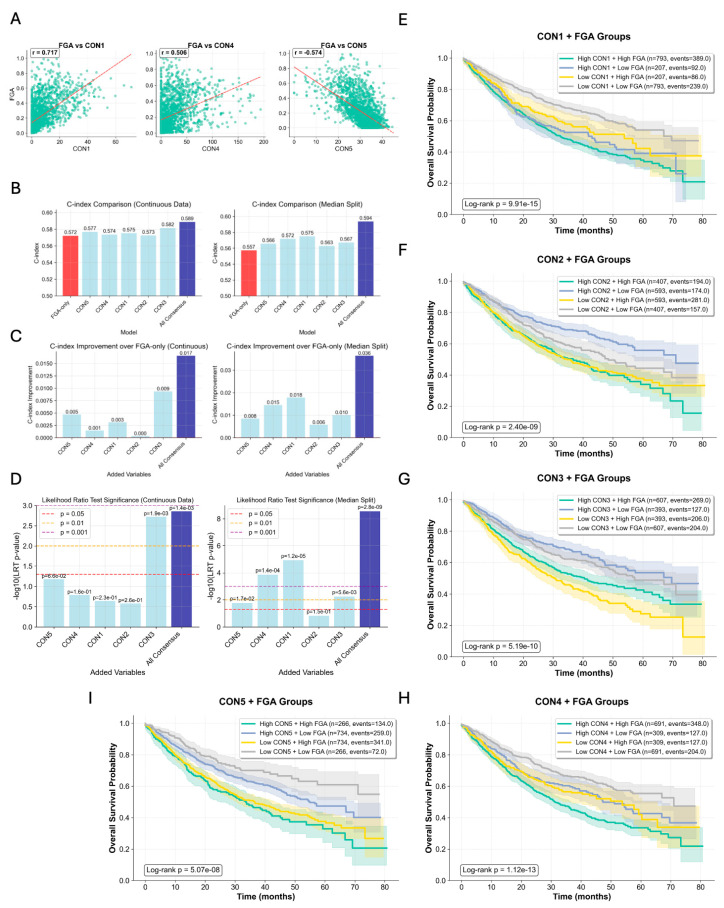
Consensus signatures provide prognostic value beyond the Fraction of Genome Altered (FGA). (**A**) Scatter plots showing Spearman’s correlation between the activity of representative signatures (CON1, CON4, CON5) and FGA. (**B**) Comparison of prognostic power using C-index for models containing FGA-only, each individual signature, and all signatures combined. (**C**) Bar plots quantifying the improvement in C-index when adding individual signatures or the full consensus model to an FGA-only baseline model. (**D**) Statistical significance (−log10 *p*-value) from the Likelihood Ratio Test, comparing the fit of the FGA-only model to models incorporating the consensus signatures. (**E**–**I**) Kaplan–Meier analyses demonstrating the ability of the signatures to further stratify patient outcomes within FGA-defined risk groups (e.g., high CON1/high FGA vs. low CON1/high FGA). The colored shading represents the 95% confidence intervals.

Given the established prognostic significance of chromosomal instability in cancer [1,20], we investigated whether our consensus CNA signatures provide additional prognostic value beyond traditional measures of genomic instability. Using the MSK-IMPACT sarcoma cohort, we analyzed the relationship between consensus signatures and Fraction of Genome Altered (FGA), a well-validated measure of overall chromosomal instability, associated with overall survival (OS) [21]. Our consensus signatures showed varying degrees of correlation with FGA. Consensus signatures 1 and 4 demonstrated strong and moderate positive correlations with FGA (Spearman correlation: 0.71 and 0.51, respectively), indicating their association with high chromosomal instability (Figure 5A). In contrast, consensus signature 5 showed a moderate negative correlation with FGA (−0.57), suggesting its association with chromosomally stable tumors (Figure 5A). CON2 and CON3 showed no significant association with FGA. When evaluated as continuous and dichotomic variables in multivariable Cox regression models controlling for FGA, the combination of all five consensus signatures significantly improved prognostic performance over FGA alone (concordance index [C-index] improvement: 0.017, *p* = 0.001) (Figure 5B). Individual consensus signatures also contributed meaningfully, with CON3 showing the strongest independent effect (C-index improvement: 0.009, *p* = 0.002) (Figure 5C,D). Median split survival analysis revealed that consensus signatures maintain prognostic value across different FGA levels (Figure 5B–D). The combined model using dichotomized variables showed even greater improvement over FGA alone (C-index improvement: 0.036, *p* < 1 × 10^−8^). Individual signatures demonstrated variable but significant contributions, with consensus signatures 1, 3, and 4 showing the strongest effects (*p* < 0.001 for each). Kaplan–Meier analysis of combined signature-FGA groups demonstrated clear survival stratification. Patients with low CON1 and low FGA had the best OS compared with all other groups, with low CON1 high FGA second best, indicating a stronger stratification of CON1 than FGA (Figure 5E). In CON2 analysis, FGA seemed to be a stronger stratifier; however, within the low FGA groups, low CON2 had better OS than high CON2, providing better stratification than using only FGA (Figure 5F). A similar pattern was observed with CON3: Within FGA high samples, patients with high CON3 had better OS than low CON3 (Figure 5G). CON4 also provided important stratification: High CON4 high FGA had the worst OS, low CON4 high FGA had the best OS, and no OS differences were observed in the mixed groups (Figure 5H). Lastly, CON5 provided a moderate stratification beyond FGA (Figure 5I). These findings demonstrate that consensus CNA signatures capture biologically meaningful patterns of chromosomal alterations that extend beyond simple measures of genomic instability. The signatures provide independent prognostic information that enhances OS stratification when combined with traditional chromosomal instability metrics, supporting their potential clinical utility as refined biomarkers for patient stratification.

### 2.7. Genomic Landscape of Consensus CNA Signatures

Next, the genomic landscape of mutations and gene-level amplifications/deletions within the CON signatures was characterized (Section 4). In an analysis of 23,712 cancer samples with both CNA profiles and mutational profiles, distinct genomic alteration patterns associated with each of the five consensus CNA signatures were revealed, identifying 859 statistically significant associations (FDR < 0.05) between genetic alterations and signature activities, comprising 614 mutation associations and 245 gene-level CNA associations. Additionally, signature-specific enrichment analysis revealed 474 significant enrichments, providing complementary insights into the molecular characteristics distinguishing each signature.

**Figure 6:**
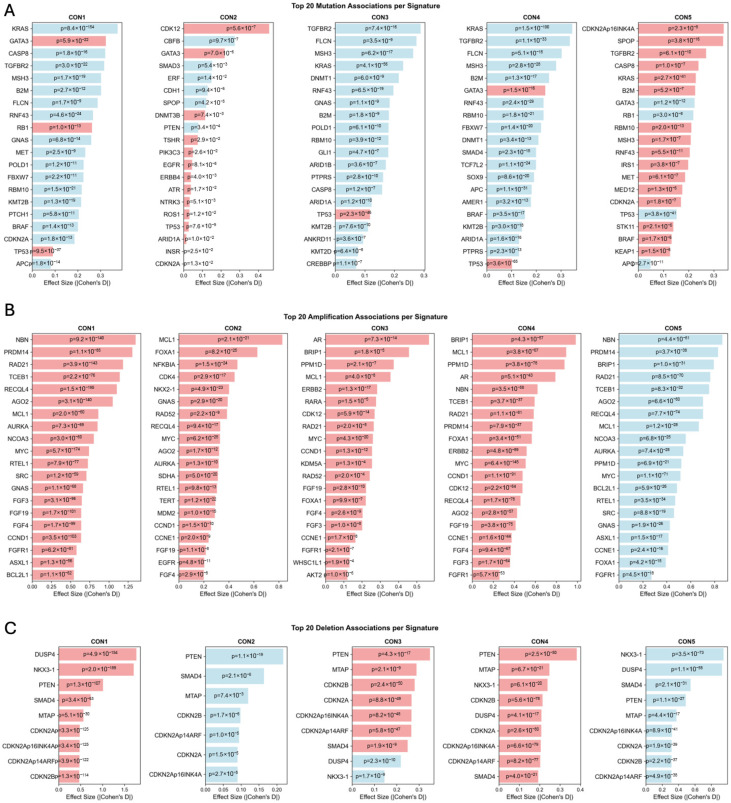
Genomic correlates of consensus CNA signatures. Bar plots illustrating the top significant associations between signature activities and the landscape of somatic alterations. Red bars indicate positive associations (enrichment); blue bars indicate negative associations (depletion). The analysis shows the effect size (Cohen’s D) and statistical significance (FDR-corrected *p*-value) for associations between each of the five signatures and (**A**) somatic mutations in cancer-related genes, (**B**) gene amplifications, and (**C**) gene deletions. These analyses connect the statistically derived signatures to underlying biological pathways and driver events.

Importantly, the consensus CNA signatures were derived exclusively from copy-number data, independent of mutational information. The following analyses characterize mutation co-occurrence patterns associated with each signature, representing correlations rather than causal relationships. Each consensus signature exhibited characteristic mutation patterns reflecting different oncogenic processes (Figure 6A). In general, as expected, CON1–4 were mostly associated with depletion of mutations relative to CON5, which is associated with a relatively diploid genome that might need more mutations to gather oncogenic processes. However, the analysis revealed distinct associations. For example, KRAS mutations were depleted across CON 1, 3 and 4 (*p* < 1 × 10^−55^), while strongly positively associated with CON5 (*p* < 1 × 10^−40^). In contrast, TP53 was largely associated with a chromosomal unstable genome and was depleted in CON5. Strikingly, GATA3 and RB1 were also strongly depleted in CON5 and highly enriched in CON 1, 2 and 4. CON2 was also positively associated with mutations such as CDK12, EGFR, ERBB4, ROS1, ATR and NTRK3. In a cancer-specific analysis, global patterns remain while more subtle patterns were discovered (Supplementary Figures S4 and S5). In breast cancer, CON1 was associated with mutations in ESR1, BRCA2 and ATRX among others, while CON5 was associated with PIK3CA and CDH1 mutations. In NSCLC, many gene mutations were enriched in CON1–4, including EGFR in CON 1, 2 and 4; PTPRT and PTPRD in CON 1, 3 and 4; ERBB4 in CON 2 and 3; and PTEN in CON 1 and 4. In CRC, there was a modest inverse pattern: Almost no mutations were enriched in CON1–4 except TP53, while many mutations were enriched in CON5, including: the mismatch repair genes MSH2, MSH3 and MSH6; POLE mutations; and NOTCH1-4 and TGF-related genes. In prostate cancer, notably, CDK12 mutations were highly enriched in CON2–4 with large effect sizes (0.45–1.4), while depleted in CON1 and CON5. In CON1, mutations in hormone-related genes were enriched, like AR, and BRCA2 was also enriched. BRCA2 mutations were also enriched in CON1 pancreatic cancer (Supplementary Figure S4).

Similarly, gene-level CNA analysis (i.e., gene amplification or deletion) revealed signature-specific amplification and deletion patterns that complement the mutational landscape (Figure 6B). CON1 showed significant enrichment for oncogene amplifications, including MYC (effect size = 0.74, FDR = 5.1 × 10^−172^), CCND1 (effect size = 0.64, FDR = 6.7 × 10^−102^), and multiple receptor tyrosine kinases. Conversely, CON5 was consistently depleted for these same amplifications, with significant depletion for CCND1, ERBB2, and FGFR1. Tumor suppressor deletions showed signature-specific patterns, (Figure 6C) with CON1 enriched for CDKN2A deletions (effect size = 0.48, FDR = 9.3 × 10^−124^) and PTEN deletions (effect size = 1.01, FDR = 2.7 × 10^−106^). These patterns suggest that different signatures capture tumors with distinct mechanisms of growth control dysregulation.

### 2.8. CNA Signatures Associated with Response and Resistance to Therapy

**Figure 7:**
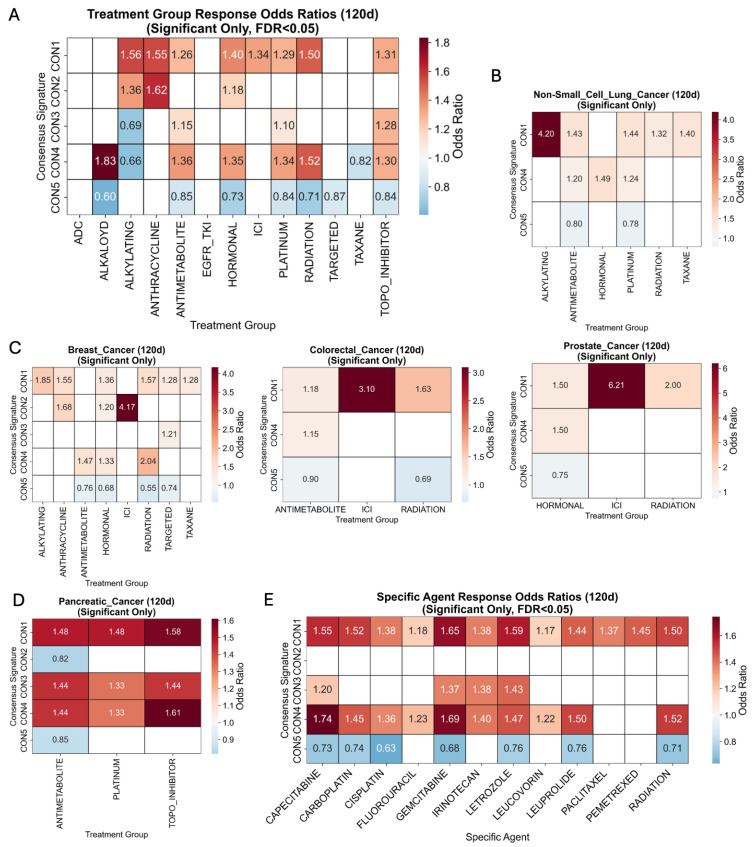
Association of consensus CNA signatures with response and resistance to systemic therapies. Odds ratios (ORs) were calculated for therapy response, defined as the absence of progressive disease at 120 days post-treatment initiation. An OR > 1 suggests an association with treatment resistance, while an OR < 1 suggests an association with treatment sensitivity. Only statistically significant associations (FDR < 0.05) are displayed. (**A**) Pan-cancer ORs for response to treatment groups. (**B**–**D**) Cancer type-specific ORs for response to major treatment classes, including chemotherapy, hormonal therapy, and immune-checkpoint inhibitors (ICIs). (**E**) Pan-cancer ORs for response to specific therapeutic agents.

Briefly, the MSK-CHORD dataset consists of NLP-based annotations of treatments and progression along the patient timeline [19]. Using 120 days as a cutoff, each patient–drug pair has a progression annotation of progression, no progression, or unclear (Section 4). An integrated analysis of 80,000 treatment events across the cohort demonstrated that CON1–5 signatures are associated with therapeutic outcome (Supplementary Figures S6–S9). Signatures CON4 and CON1 exhibited the broadest resistance spectra, with elevated 120-day progression under platinum-based treatments, antimetabolites, radiation therapy and hormonal therapy both in pan-cancer and cancer-specific analyses (CON4: 6 drug classes, 10 agents; CON1: 8 classes, 12 agents) (Figure 7A–E). Crucially, CON1 also showed marked refractoriness to immune-checkpoint inhibition (ICI): at the class level, CON1 carried a significant ICI odds ratio (OR) of 1.34 (95% CI 1.10–1.64, FDR = 0.010), while tumor-stratified analyses revealed even stronger effects: prostate cancer OR = 6.17, FDR = 0.039, and colorectal cancer OR = 3.10, FDR = 0.032 (Figure 7C). CON1 resistance pattern extended to radiation therapy (OR = 1.50, *p* < 0.001) across all cancer types, with particularly pronounced effects in prostate cancer (OR = 2.00, *p* < 0.001). Additionally, in breast cancer, CON2 was associated with ICI resistance (OR = 4.17, FDR = 0.010) (Figure 7C). In contrast, the genomically quiescent CON5 was uniformly sensitive, displaying reduced progression across seven drug classes, including ICIs (class OR = 0.84), and across all five tumor types examined. Intermediate phenotypes emerged for CON3, which retained alkylator sensitivity (OR = 0.69) despite resistance to platinum and topoisomerase inhibition, and for CON2, whose resistance was largely confined to endocrine, anthracycline and ICI therapy.

To explore the entire signature landscape of each patient, multi-signature vector analysis using Hotelling’s T^2^ tests was performed. This demonstrated significant multivariate differences between treatment responders and non-responders (T^2^ = 316.68, *p* < 1 × 10^−16^), with Linear Discriminant Analysis (LDA) achieving 73.33% classification accuracy (See Section 4; Supplementary Figures S6 and S7). Furthermore, machine learning (ML) models were trained to predict 120-day response using the signatures and clinical parameters as features. Features included were the five consensus signature values (CON1–CON5), clinical variables (age, sex, mutation count, stage, HER2 status), and 30 engineered features encompassing signature ratios, interactions, and statistical summaries (Section 4). The models achieved clinically meaningful prediction performance on the validation set, with XGBoost attaining 79.74% AUC and 92.51% average precision (Supplementary Figures S8 and S9). Feature importance using SHapley Additive exPlanations (SHAP) analysis revealed that treatment agent type, patient age, and signature variability were the most predictive factors, while signature interactions and ratios proved more informative than individual signature values (Supplementary Figure S9).

## 3. Discussion

This study demonstrates that clinically acquired targeted panel data, despite covering <2% of the genome, can yield biologically meaningful and clinically actionable CNA signatures when analyzed with a consensus, multi-algorithm framework. Using four complementary methods, we extracted five reproducible signatures (CON1–CON5) from 24,870 MSK-IMPACT profiles and validated them across >2000 additional tumors. These signatures retained independent prognostic power across diverse histologies, outperformed single-algorithm models and complemented established measures of chromosomal instability. These findings extend the concept of mutational signatures beyond whole WGS and bring CNA-based biomarkers within reach of routine precision oncology.

The signatures map onto discrete genomic processes. CON4 is enriched for TP53 loss and broad chromosomal gains, consistent with mitotic checkpoint failure; CON3 comprises arm-level loss of heterozygosity and tracks with homologous-recombination deficiency-like profiles; and CON5 segregates with KRAS mutation and mismatch repair gene disruption, suggesting a link between RAS signaling, replication stress and segmental LOH. Importantly, these patterns translate into therapy-relevant phenotypes: CON1 and CON4 were associated with resistance to platinum-based chemotherapy, antimetabolites and immune-checkpoint blockade, whereas CON5 marks relative drug sensitivity across multiple classes. Such associations could inform trial stratification, for example, testing whether CON1 high colorectal cancers benefit from intensified chemotherapy or novel DNA-damage response inhibitors. The association between high CNA burden and immune-evasion-driven ICI failure accords with prior work linking aneuploidy to impaired antigen presentation and T-cell exclusion [22,23]. Specifically, large-scale LOH events, the hallmark of CON1, may directly reduce neoantigen burden by eliminating mutant alleles [24]. The genomic complexity inherent to these signatures may also confer adaptive capacity, enabling resistance to cytotoxic therapies through clonal heterogeneity [25]. These hypotheses warrant functional validation in model systems.

Because MSK-IMPACT and comparable 300–500 gene panels are deployed in >100,000 patients annually, fitting CON signatures requires only re-analysis of data already generated in routine care. The non-negative least squares model runs in <1 s per sample on a laptop and attained 93% variance capture, facilitating prospective use. The addition of signature burden improved survival stratification beyond fraction genome altered (FGA) and traditional clinicopathological covariates, underscoring their complementary information content. Integrating signature calls into automated clinical reports could therefore enable real-time risk stratification and inform multidisciplinary treatment planning. For example, molecular tumor boards, often discussing patients with complex genomic and clinical trajectories, could incorporate additional prognostic and therapeutic insights if CON signatures were labeled.

The presented work has several limitations. First, the discovery and validation cohorts were sequenced on the same platform within one institution; multi-center studies and other panel designs will be required to confirm generalizability. Second, the public MSK datasets lack LOH annotation, forcing exclusion of those events. Future releases with an allele-specific copy-number could refine signature definitions. Third, survival analyses are retrospective and susceptible to residual confounding. Finally, treatment response findings derive from heterogeneous real-world endpoints and warrant confirmation in controlled trials. Additionally, while bootstrap stability analysis demonstrated robust consensus signatures (mean stability 0.70, with CON4 and CON5 achieving stability > 0.95), validation on non-MSK panels using different gene sets and platforms remains necessary to establish cross-platform generalizability. Furthermore, signature feature values represent the fraction of detected CNA segments within the panel’s coverage area, and interpretation should account for the coverage of MSK-IMPACT. To adapt the framework for alternative panels, users would need to: (1) map panel-specific CNA calls to the 28-feature classification scheme using segment coordinates; (2) optionally re-derive consensus signatures if the panel’s gene content differs substantially from MSK-IMPACT; or (3) apply the existing CON signatures directly if coverage is comparable, accepting that panel-specific biases may attenuate effect sizes. The modular design of our pipeline facilitates such adaptations.

Therefore, prospective studies embedding CON signature reporting into tumor boards could establish clinical impact and cost-effectiveness. Algorithmically, joint modeling of SNV, indel and structural-variation signatures may reveal composite genomic phenotypes that may better predict therapy response. Functionally, CRISPR or organoid models stratified by signature could dissect mechanistic underpinnings—particularly the interplay between genomic mutations and CNA signatures. Extending the pipeline to liquid-biopsy panels may enable non-invasive monitoring of CNA signature dynamics during treatment.

To conclude, by showing that sparse panel data can be transformed into robust CNA signatures with prognostic and therapeutic relevance, this work might bridge a translational gap between research-grade genomics and oncology practice. The open-source consensus framework provides a scalable foundation for future biomarker discovery, clinical implementation and mechanistic exploration of chromosomal instability in cancer.

## 4. Materials and Methods

### 4.1. CNA Signature Analysis

First, raw CNA segments were classified into 28 classes: (1) Homozygous deletions, 3 length classes: 0–100 Kb, 100 Kb–1 Mb, above 1 Mb basepairs. (2) Heterozygous deletion, diploid, and amplification. Four copy-number classes, 5 length classes in each. Copy-number classes: 1 copy (LOH), 2 copies, 3–4 copies, 5–8 copies, and above 9 copies. Length classes: 0–100 Kb, 100 Kb–1 Mb, 1–10 Mb, 10–40 Mb, and above 40 Mb basepairs. The general scheme is based on Khandekar et al. [26]. For every category, we recorded the fraction of the genome affected, producing a 24,870 × 28 non-negative matrix. This matrix served as the input for four different deconvolution algorithms and frameworks: (1) Non-NMF, utilizing the state-of-the-art framework SigProfilerExtractor v1.1 [27] with default parameters. (2) Hierarchical Dirichlet process, utilizing the state-of-the-art framework mSigHdp v2.1.2 [28] with default parameters. (3) ICA, a computational method for separating a multivariate signal into additive subcomponents that are maximally independent. Implemented using Python 3.12 via the FastICA and NMF modules in the scikit-learn package v1.7.0. The non-negative ICA approach combines NMF initialization with iterative orthogonalization to enhance component independence while maintaining non-negativity constraints. The parameters were optimized through cross-validation based on reconstruction error and component independence metrics. Signature quality was assessed using reconstruction accuracy, mean absolute correlation between components, and neighborhood preservation in the transformed space. (4) Graph-Regularized CNA Signature Extraction. Implemented using Python 3.12 via the networkx package v3.4. Briefly, the graph is constructed based on a similarity matrix, where the nodes are the CNA categories, the edges are the correlation strength between categories, and the weights are the absolute correlations values. Graph Laplacian constructs the regularization matrix. Then, a Graph-NMF is implemented using Python 3.12. The Graph-NMF incorporates relationships between CNA categories through a correlation-based graph structure. The method decomposes the CNA matrix while enforcing graph regularization that encourages similar CNA categories to exhibit similar signature patterns, along with diversity and sparsity penalties. The graph Laplacian regularization term preserves local neighborhood structures in the CNA feature space, while diversity constraints prevent redundant signatures and sparsity penalties focus signatures on key genomic features. Signature stability was assessed through multiple random initializations and measured using cross-run correlations and activity clustering metrics.

### 4.2. Consensus CNA Framework

The consensus approach integrates CNA signatures from the four complementary methods. All signatures were L1-normalized and pairwise cosine similarities computed across all method-derived signatures. Hierarchical clustering with Ward linkage was applied to the resulting similarity matrix, with optimal cluster number determined by maximizing the proportion of clusters meeting validation criteria, as follows:

Similarity assessment and clustering: Pairwise signature similarities were computed using cosine similarity, chosen for its scale invariance and suitability for compositional data. Hierarchical clustering with Ward linkage was applied to group similar signatures, with optimal cluster number determined through adaptive scoring that maximized the ratio of valid clusters to total clusters tested (range: 4–15 clusters).

Cluster validation: Clusters were validated using flexible multi-criteria logic requiring satisfaction of at least one condition: (1) cross-method diversity (≥2 different methods), (2) proportional representation (≥26% of total methods, equivalent to substantial contribution from at least one method), or (3) method concentration (≥3 signatures from a single method). This framework accommodated both conserved signatures identified across multiple methods and method-specific robust patterns.

Consensus derivation: Within each validated cluster, consensus signatures were derived as the unweighted median across constituent signatures. Median aggregation was chosen for its robustness to outliers and non-parametric properties, avoiding distributional assumptions while ensuring equal methodological contribution. The final consensus comprised five signatures representing distinct CNA patterns.

Stability: Signature stability was assessed through bootstrap resampling (*n* = 100 iterations, 80% subsampling) measuring correlations between original and resampled consensus signatures. Additional validation included silhouette analysis for cluster quality (mean silhouette width), cophenetic correlation for hierarchical clustering faithfulness, and reconstruction error assessment using non-negative least squares regression.

The consensus framework demonstrated strong hierarchical representation (cophenetic correlation 0.66), and, not surprisingly, modest cluster coherence (silhouette score 0.29). Mean methods per cluster: 2.4, demonstrating method diversity. The bootstrap analysis demonstrated good overall stability (mean bootstrap correlation = 0.70).

### 4.3. Patient Cohorts

Discovery set: 24,870 primary and metastatic tumors from 5 different cancer types, consecutively sequenced in the Memorial Sloan Kettering Integrated Mutation Profiling of Actionable Cancer Targets (MSK-IMPACT) program [19]. The data consist of clinical annotation such as age, sex, primary site, stage at sequencing, systemic treatments, Natural Language Processing (NLP)-based response to treatments, overall survival status, and genomics data including processed CNA profiles at a gene level and at a chromosomal length level. There are no data regarding LOH, and deep deletions are also almost not reported due to the method’s limitations.

External validation sets: Sarcoma (MSK-SARC) [29]: 1464 tumors; hepatocellular cancer (MSK-HCC) [30]: 288 tumors. Both cohorts were sequenced prospectively with the MSK-IMPACT and annotated independently of the discovery dataset.

### 4.4. Survival Analysis

Survival analyses were conducted using Kaplan–Meier estimation with log-rank testing, stratifying patients either by a continuous measuring of activity, grouping into high and low activity groups using median-based stratification, using quantile-based stratification, or by tertile splits (top 33% versus bottom 33% of signature activity). To assess cross-cancer generalizability, analyses were performed both overall and stratified by major cancer types (non-small cell lung cancer, breast cancer, colorectal cancer, prostate cancer, and pancreatic cancer) with minimum sample size requirements of 100 patients per cancer type. Performance metrics included statistical significance (log-rank *p*-values), survival curve separation (24-month survival differences), and signature success rates (proportion of statistically significant signatures per method).

### 4.5. CNA Signature Fitting in New Samples

We developed a comprehensive computational framework for fitting copy-number alteration (CNA) signatures to independent cancer genomic datasets. The approach utilizes a two-stage pipeline: first, raw cbioportal segmentation files were processed and converted to CNV feature matrices using the established CNV28 framework, which quantifies 28 distinct copy-number features including amplifications, deletions, and chromosomal instability patterns. Then, we applied non-negative least squares (nnls) to decompose the CNV28 matrices against the pre-defined consensus CNA signatures, yielding signature activities for each sample. The fitting algorithm employs constrained optimization with L2 regularization to ensure numerical stability. Technical validation was assessed using coefficient of determination (R^2^) and reconstruction error metrics, while clinical validation employed Cox proportional hazards regression and Kaplan–Meier survival analysis to evaluate prognostic associations. The complete pipeline was implemented as a one-click solution.

### 4.6. Genomic Correlates of Signatures

Consensus CNA signature activities were systematically integrated with genomic alteration data and gene-level amplifications/deletions to identify signature-specific genetic alterations. Mutation data were processed and converted to binary matrices (mutated = 1, wild-type = 0), focusing on genes mutated in ≥1% of samples. For gene-level copy-number alterations, amplifications were defined as scores ≥ 1 and deletions as scores ≤ −1. Two complementary analytical approaches were employed: (1) Association analysis compared signature activities between altered versus wild-type samples using Mann–Whitney U tests with Cohen’s d for effect size quantification. (2) Enrichment analysis identified signature-specific alterations by comparing high-activity samples for each signature against high-activity samples of all other signatures using Fisher’s exact tests. All analyses employed Benjamini–Hochberg false discovery rate (FDR) correction for multiple testing, with significance defined as FDR < 0.05 and meaningful biological effects filtered by |Cohen’s d| > 0.2 for associations and fold enrichment > 1.2 for enrichment analysis.

### 4.7. Treatment Response Analysis

Treatment response associations were systematically evaluated using MSK-CHORD treatment records [19], employing three complementary analytical approaches. Univariate analysis compared 120-day progression rates between high- and low-consensus signature activity groups (median split) using Fisher’s exact tests with Benjamini–Hochberg FDR correction (*p* < 0.05). Treatment groups included chemotherapy classes, radiation (stage 1–3 only), hormonal therapy, immunotherapy, and targeted therapy. In addition, agent-specific analysis with 52 different therapeutic agents was performed. Multivariable machine learning analysis implemented both traditional ML (XGBoost, LightGBM, Random Forest) and neural networks deep learning models for treatment response prediction. Features included the 5 consensus signature values (CON1–CON5), clinical variables (age, sex, mutation count, stage, HER2 status), and 30 engineered features encompassing signature ratios, interactions, and statistical summaries. Models were trained using 80/20 train–test splits with 5-fold cross-validation. Additionally, multi-signature vector analysis treated the complete 5-signature profile as a vector input using Hotelling’s T^2^ tests for multivariate mean differences, linear/quadratic discriminant analysis for classification, and principal component analysis for dimensionality reduction. Feature importance analysis was performed using SHAP.

### 4.8. Statistical and Computational Analyses

All statistical analyses were performed using Python 3.12 with a scientific computing stack. Data manipulation and preprocessing utilized pandas 2.2.3 and NumPy 2.1.3, while statistical testing employed SciPy with corrections for multiple comparisons via statsmodels (Benjamini–Hochberg FDR). Survival analyses were conducted using the lifelines package for Kaplan–Meier estimation and Cox proportional hazards regression. Machine learning models were implemented using scikit-learn 1.7.0, XGBoost 3.0.2, and TensorFlow 2.19.0. Multivariate statistical analyses included Hotelling’s T^2^ tests and discriminant analysis, with model interpretation via SHAP values. All visualizations were generated using matplotlib and seaborn. All statistical significance was defined as *p* < 0.05 or FDR-corrected *p* < 0.05 where multiple testing corrections applied.

## Data Availability

The original data presented in the study are openly available in cbioportal (https://www.cbioportal.org/study/summary?id=msk_chord_2024, accessed on 7 November 2024). Analysis results are available in the manuscript and its Supplementary Information. Code to create the consensus signatures, code to fit the signatures in new samples, and an online platform to use the fitting framework are available in GitHub https://github.com/Adarya/CON-SIG (accessed on 9 February 2026) and in https://consig.streamlit.app/.

## Supplementary Materials

The following supporting information can be downloaded at: https://www.mdpi.com/article/10.3390/ijms27041764/s1.

## References

[1] Beroukhim R., Mermel C.H., Porter D., Wei G., Raychaudhuri S., Donovan J., Barretina J., Boehm J.S., Dobson J., Urashima M. (2010). The Landscape of Somatic Copy-Number Alteration across Human Cancers. Nature.

[2] Taylor A.M., Shih J., Ha G., Gao G.F., Zhang X., Berger A.C., Schumacher S.E., Wang C., Hu H., Liu J. (2018). Genomic and Functional Approaches to Understanding Cancer Aneuploidy. Cancer Cell.

[3] Zack T.I., Schumacher S.E., Carter S.L., Cherniack A.D., Saksena G., Tabak B., Lawrence M.S., Zhang C.-Z., Wala J., Mermel C.H. (2013). Pan-Cancer Patterns of Somatic Copy Number Alteration. Nat. Genet..

[4] Macintyre G., Goranova T.E., De Silva D., Ennis D., Piskorz A.M., Eldridge M., Sie D., Lewsley L.-A., Hanif A., Wilson C. (2018). Copy Number Signatures and Mutational Processes in Ovarian Carcinoma. Nat. Genet..

[5] Steele C.D., Abbasi A., Islam S.M.A., Bowes A.L., Khandekar A., Haase K., Hames-Fathi S., Ajayi D., Verfaillie A., Dhami P. (2022). Signatures of Copy Number Alterations in Human Cancer. Nature.

[6] Cheng M.L., Berger M.F., Hyman D.M., Solit D.B. (2018). Clinical Tumour Sequencing for Precision Oncology: Time for a Universal Strategy. Nat. Rev. Cancer.

[7] Cheng M.L., Solit D.B. (2018). Opportunities and Challenges in Genomic Sequencing for Precision Cancer Care. Ann. Intern. Med..

[8] El-Deiry W.S., Goldberg R.M., Lenz H., Shields A.F., Gibney G.T., Tan A.R., Brown J., Eisenberg B., Heath E.I., Phuphanich S. (2019). The Current State of Molecular Testing in the Treatment of Patients with Solid Tumors, 2019. CA Cancer J. Clin..

[9] Malone E.R., Oliva M., Sabatini P.J.B., Stockley T.L., Siu L.L. (2020). Molecular Profiling for Precision Cancer Therapies. Genome Med..

[10] Zehir A., Benayed R., Shah R.H., Syed A., Middha S., Kim H.R., Srinivasan P., Gao J., Chakravarty D., Devlin S.M. (2017). Mutational Landscape of Metastatic Cancer Revealed from Prospective Clinical Sequencing of 10,000 Patients. Nat. Med..

[11] Frampton G.M., Fichtenholtz A., Otto G.A., Wang K., Downing S.R., He J., Schnall-Levin M., White J., Sanford E.M., An P. (2013). Development and Validation of a Clinical Cancer Genomic Profiling Test Based on Massively Parallel DNA Sequencing. Nat. Biotechnol..

[12] Cheng D.T., Mitchell T.N., Zehir A., Shah R.H., Benayed R., Syed A., Chandramohan R., Liu Z.Y., Won H.H., Scott S.N. (2015). Memorial Sloan Kettering-Integrated Mutation Profiling of Actionable Cancer Targets (MSK-IMPACT). J. Mol. Diagn..

[13] Vives-Usano M., Pelaez B.G., Lladó R.R., Ibañez M.G., Aldeguer E., Rodriguez S., Aguilar A., Pons F., Viteri S., Cabrera C. (2021). Analysis of Copy Number Variations in Solid Tumors Using a Next Generation Sequencing Custom Panel. J. Mol. Pathol..

[14] Yaacov A., Cohen G.B., Landau J., Hope T., Simon I., Rosenberg S. (2024). Cancer Mutational Signatures Identification in Clinical Assays Using Neural Embedding-Based Representations. Cell Rep. Med..

[15] Gulhan D.C., Lee J.J.-K., Melloni G.E.M., Cortés-Ciriano I., Park P.J. (2019). Detecting the Mutational Signature of Homologous Recombination Deficiency in Clinical Samples. Nat. Genet..

[16] Wu Y., Chua E.H.Z., Ng A.W.T., Boot A., Rozen S.G. (2022). Accuracy of Mutational Signature Software on Correlated Signatures. Sci. Rep..

[17] Koh G., Degasperi A., Zou X., Momen S., Nik-Zainal S. (2021). Mutational Signatures: Emerging Concepts, Caveats and Clinical Applications. Nat. Rev. Cancer.

[18] Yang P., Yang Y.H., Zhou B.B., Zomaya A.Y. (2010). A Review of Ensemble Methods in Bioinformatics. Curr. Bioinform..

[19] Jee J., Fong C., Pichotta K., Tran T.N., Luthra A., Waters M., Fu C., Altoe M., Liu S.-Y., Maron S.B. (2024). Automated Real-World Data Integration Improves Cancer Outcome Prediction. Nature.

[20] Hieronymus H., Murali R., Tin A., Yadav K., Abida W., Moller H., Berney D., Scher H., Carver B., Scardino P. (2018). Tumor Copy Number Alteration Burden Is a Pan-Cancer Prognostic Factor Associated with Recurrence and Death. eLife.

[21] Nguyen B., Fong C., Luthra A., Smith S.A., DiNatale R.G., Nandakumar S., Walch H., Chatila W.K., Madupuri R., Kundra R. (2022). Genomic Characterization of Metastatic Patterns from Prospective Clinical Sequencing of 25,000 Patients. Cell.

[22] Davoli T., Uno H., Wooten E.C., Elledge S.J. (2017). Tumor Aneuploidy Correlates with Markers of Immune Evasion and with Reduced Response to Immunotherapy. Science.

[23] Tripathi R., Modur V., Senovilla L., Kroemer G., Komurov K. (2019). Suppression of Tumor Antigen Presentation during Aneuploid Tumor Evolution Contributes to Immune Evasion. OncoImmunology.

[24] Rosenthal R., Cadieux E.L., Salgado R., Bakir M.A., Moore D.A., Hiley C.T., Lund T., Tanić M., Reading J.L., The TRACERx Consortium (2019). Neoantigen-Directed Immune Escape in Lung Cancer Evolution. Nature.

[25] Dagogo-Jack I., Shaw A.T. (2018). Tumour Heterogeneity and Resistance to Cancer Therapies. Nat. Rev. Clin. Oncol..

[26] Khandekar A., Vangara R., Barnes M., Díaz-Gay M., Abbasi A., Bergstrom E.N., Steele C.D., Pillay N., Alexandrov L.B. (2023). Visualizing and Exploring Patterns of Large Mutational Events with SigProfilerMatrixGenerator. BMC Genom..

[27] Islam S.M.A., Díaz-Gay M., Wu Y., Barnes M., Vangara R., Bergstrom E.N., He Y., Vella M., Wang J., Teague J.W. (2022). Uncovering Novel Mutational Signatures by de Novo Extraction with SigProfilerExtractor. Cell Genom..

[28] Liu M., Wu Y., Jiang N., Boot A., Rozen S.G. (2023). mSigHdp: Hierarchical Dirichlet Process Mixture Modeling for Mutational Signature Discovery. NAR Genom. Bioinform..

[29] Nacev B.A., Sanchez-Vega F., Smith S.A., Antonescu C.R., Rosenbaum E., Shi H., Tang C., Socci N.D., Rana S., Gularte-Mérida R. (2022). Clinical Sequencing of Soft Tissue and Bone Sarcomas Delineates Diverse Genomic Landscapes and Potential Therapeutic Targets. Nat. Commun..

[30] Song Y., Boerner T., Drill E., Shin P., Kumar S., Sigel C., Cercek A., Kemeny N., Abou-Alfa G., Iacobuzio-Donahue C. (2024). A Novel Approach to Quantify Heterogeneity of Intrahepatic Cholangiocarcinoma: The Hidden-Genome Classifier. Clin. Cancer Res..

