# Supplementary material for: Consensus Copy-Number Alteration Signatures from Clinical Panels Enable Pan-Cancer Risk Stratification and Therapy Response Association
Source: Int J Mol Sci. 2026 Feb 12;27(4):1764. doi: 10.3390/ijms27041764 (PMC12940284; doi:10.3390/ijms27041764)
Supplement: Supplementary file 1 [file ijms-27-01764-s001.zip › ijms-4131213-supplementary.pdf]

Supplementary Figure S1

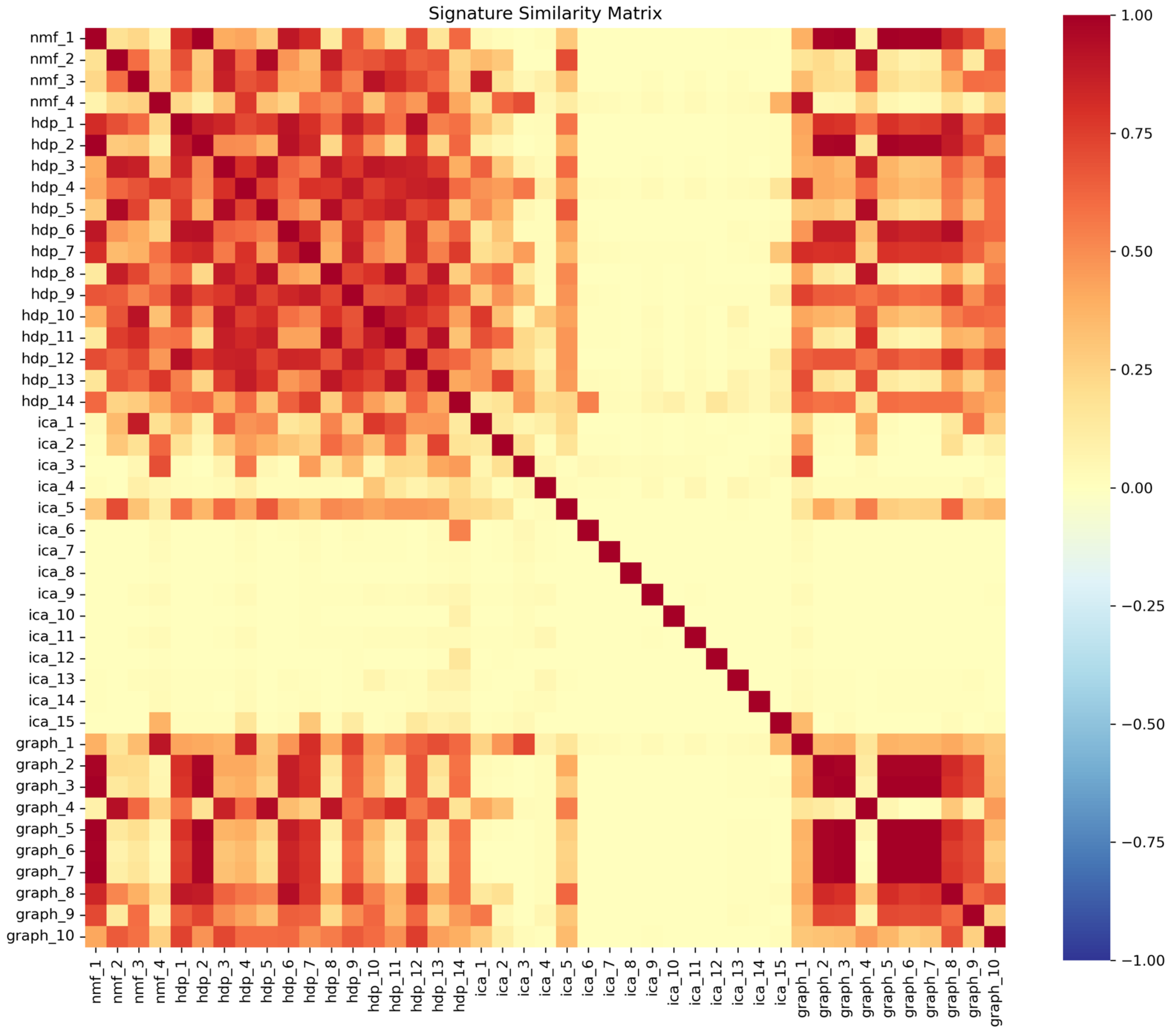

Supplementary Figure S1. Heatmap showing Cosine Similarity values across all signatures derived from 4 different methods – NMF, HDP, ICA, GD (graph)

Supplementary Figure S2

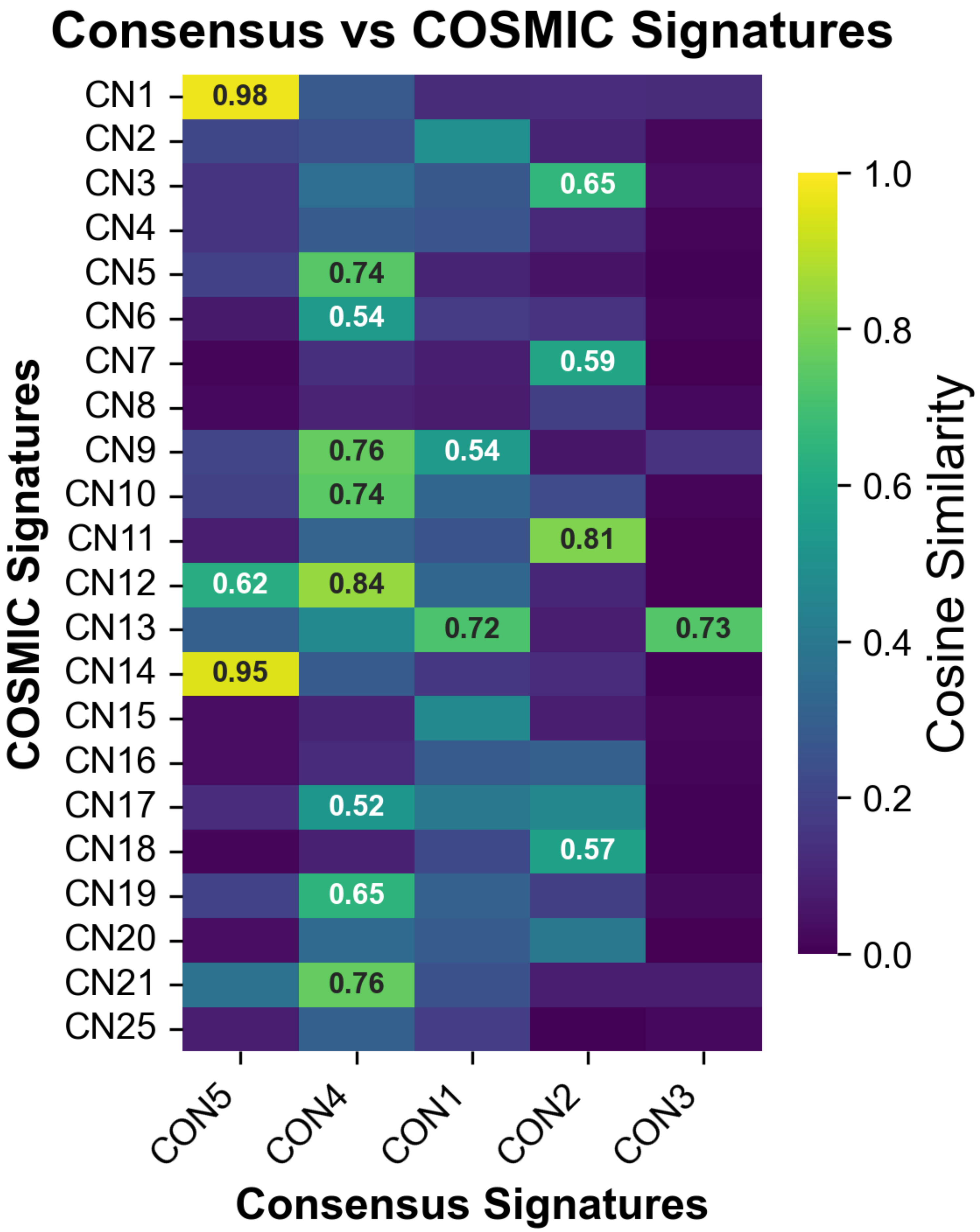

Supplementary Figure S2. Heatmap showing Cosine Similarity values of the consensus panel-based CON1-5 signatures with COSMIC's WGS/SNP-based signatures.

Supplementary Figure S3

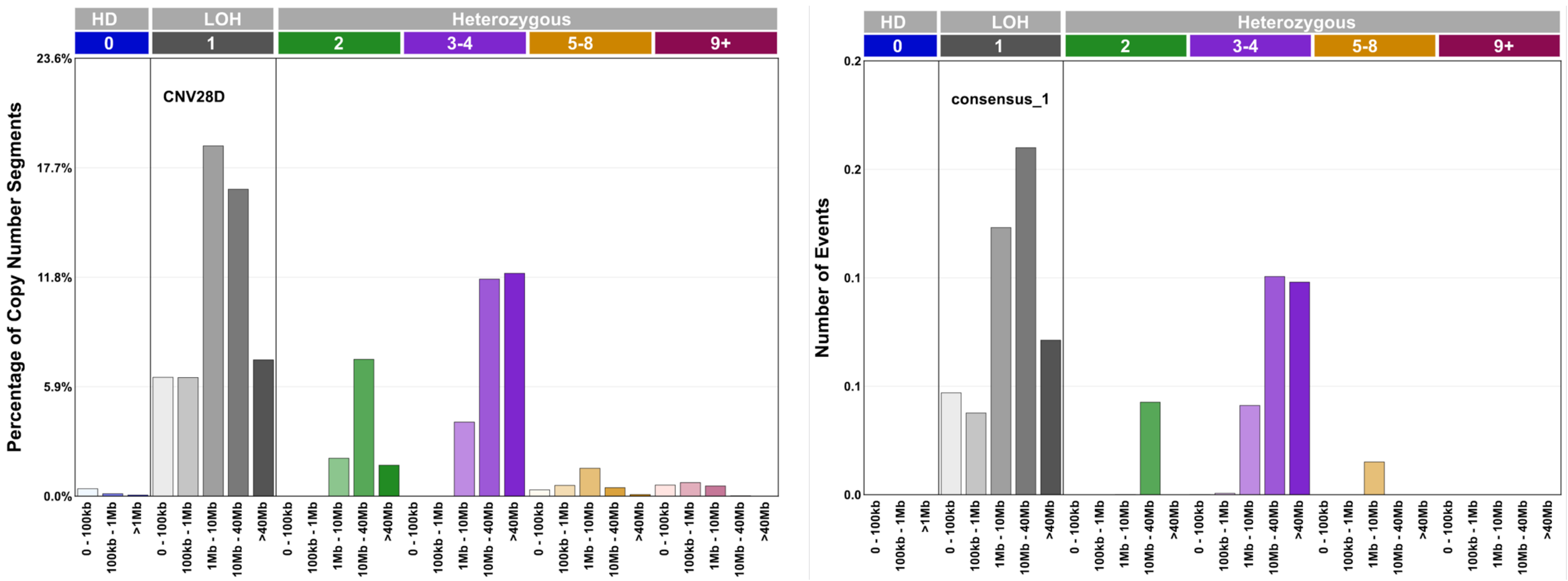

Supplementary Figure S3. Barplot showing pattern distribution of NMF-based signature D and consensus CON1 signatures side-by-side.

Supplementary Figure S4

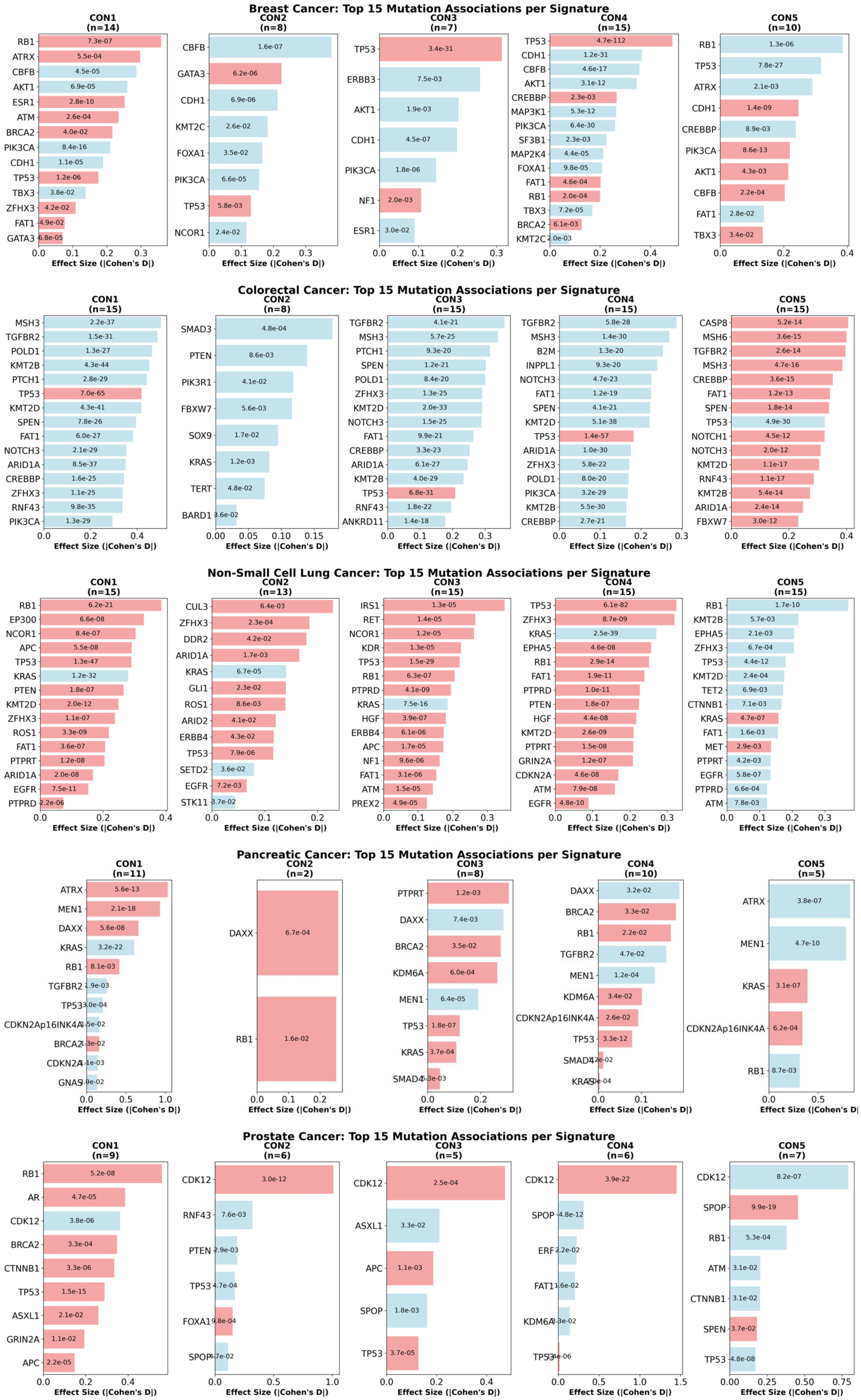

Supplementary Figure S4. Bar plots illustrating the top significant associations between signature activities and the landscape of somatic alterations in a cancer-specific manner

Supplementary Figure S5

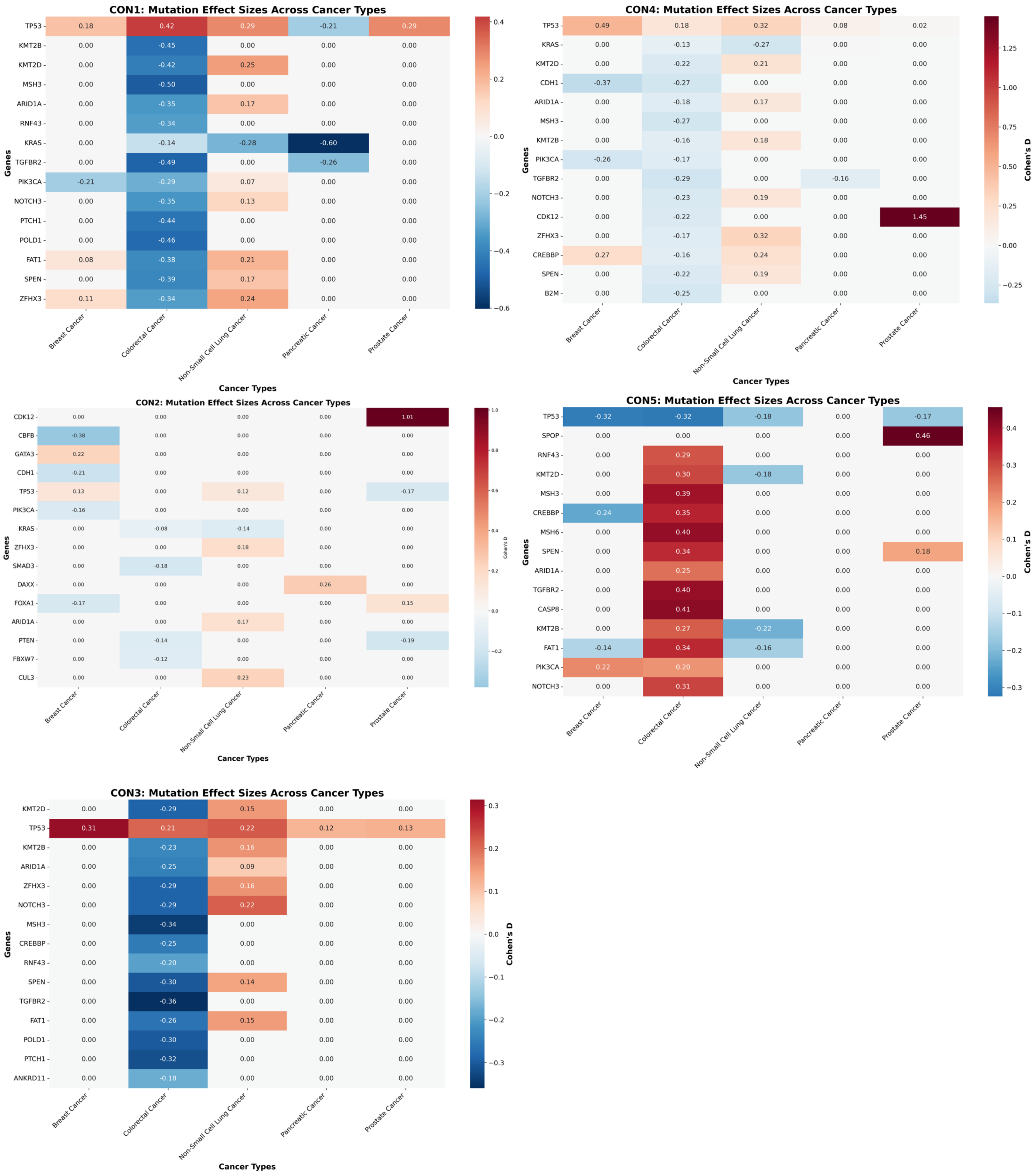

Supplementary Figure S5. Heatmaps showing Effect sizes of genomic-signature association across cancer types and different signatures.

Supplementary Figure S6

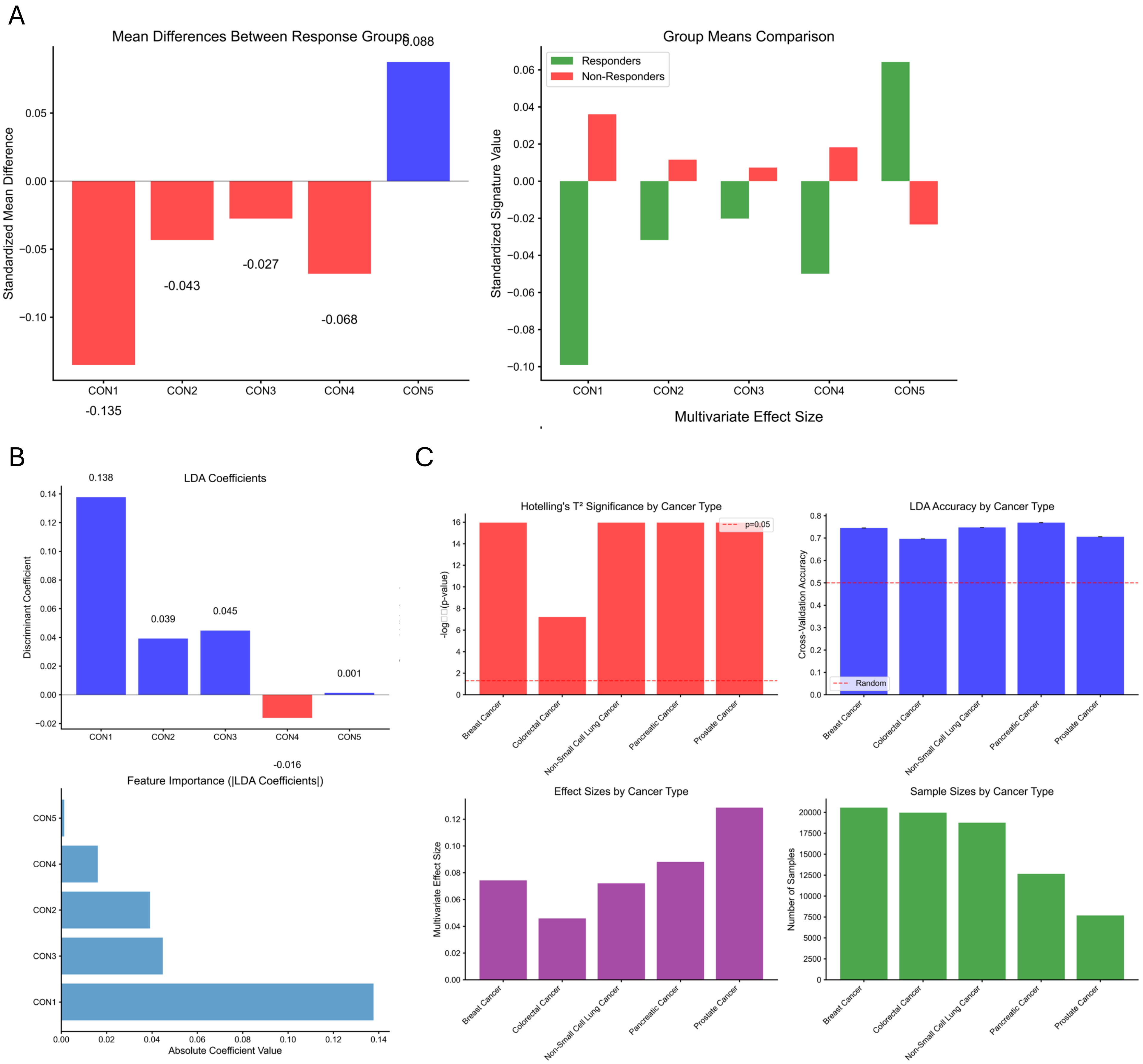

Supplementary Figure S6. response analyses results. (A) mean-based analysis between responders and non-responders. (B) LDA coefficients and importance. (C,D) Hotelling's T^2 and LDA analyses results.

Supplementary Figure S7

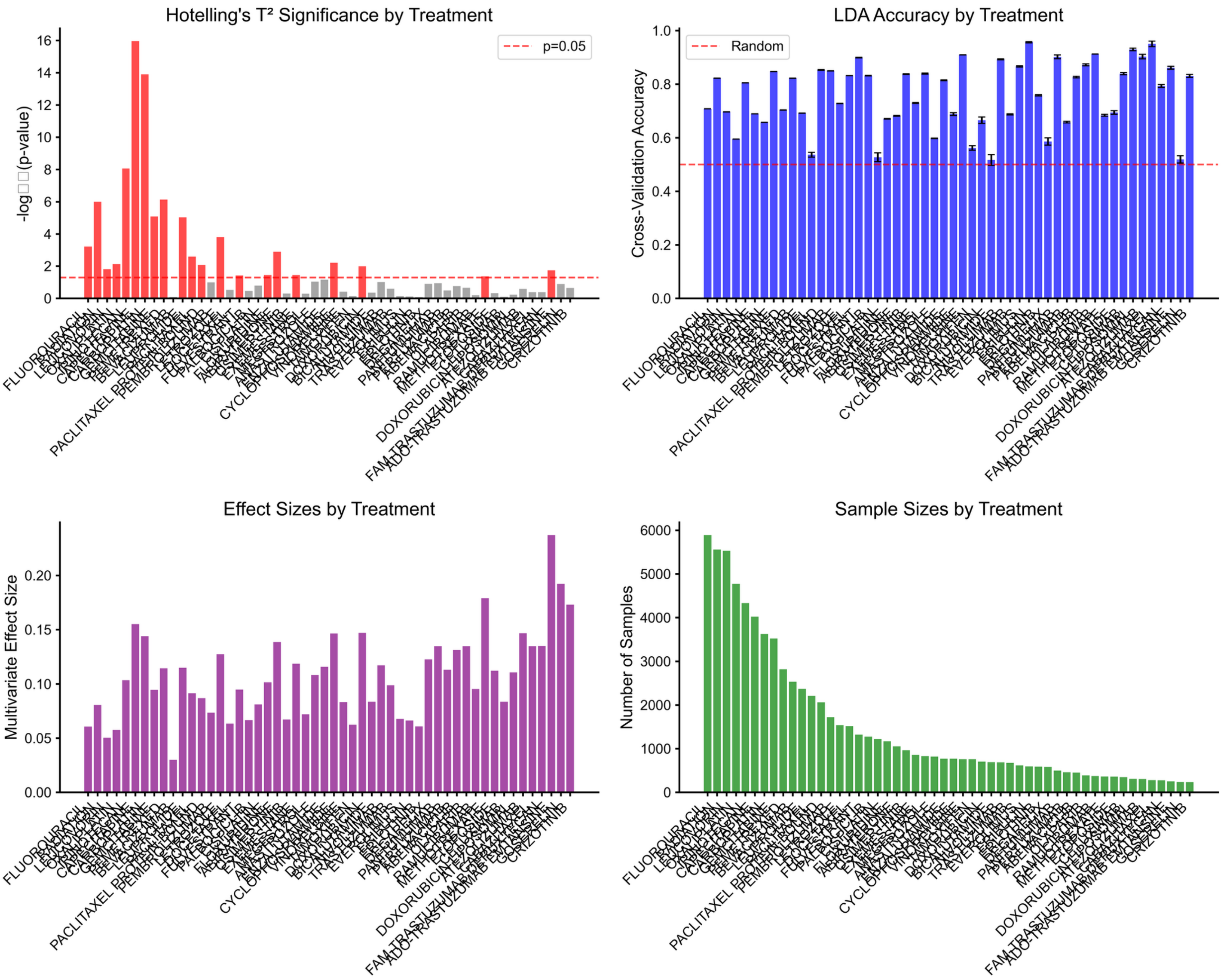

Supplementary Figure S7. Hotelling's T^2 and LDA analyses results in an agent-specific manner.

Supplementary Figure S8

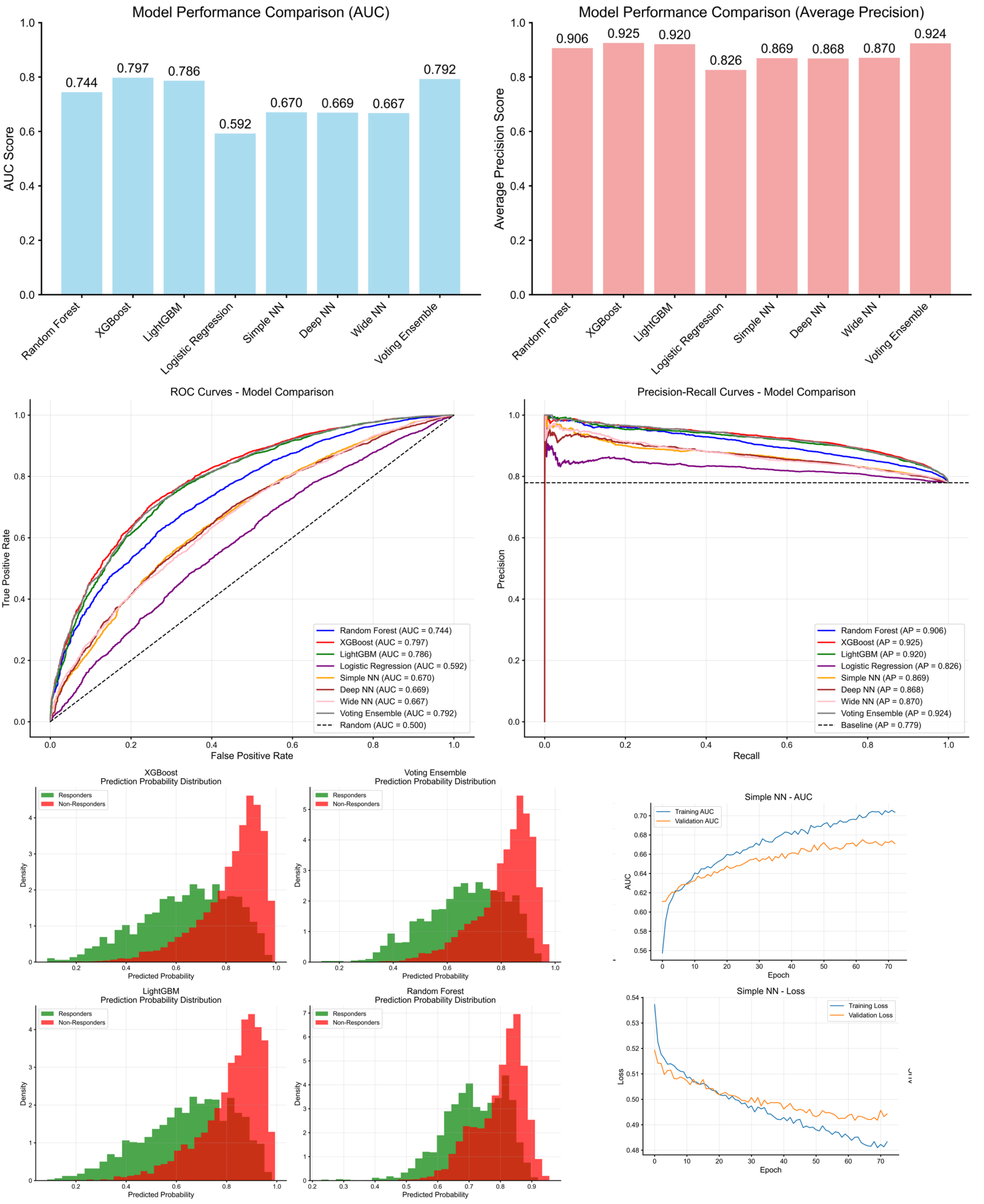

Supplementary Figure S8. Predictive models validation set scores and metrics.

Supplementary Figure S9

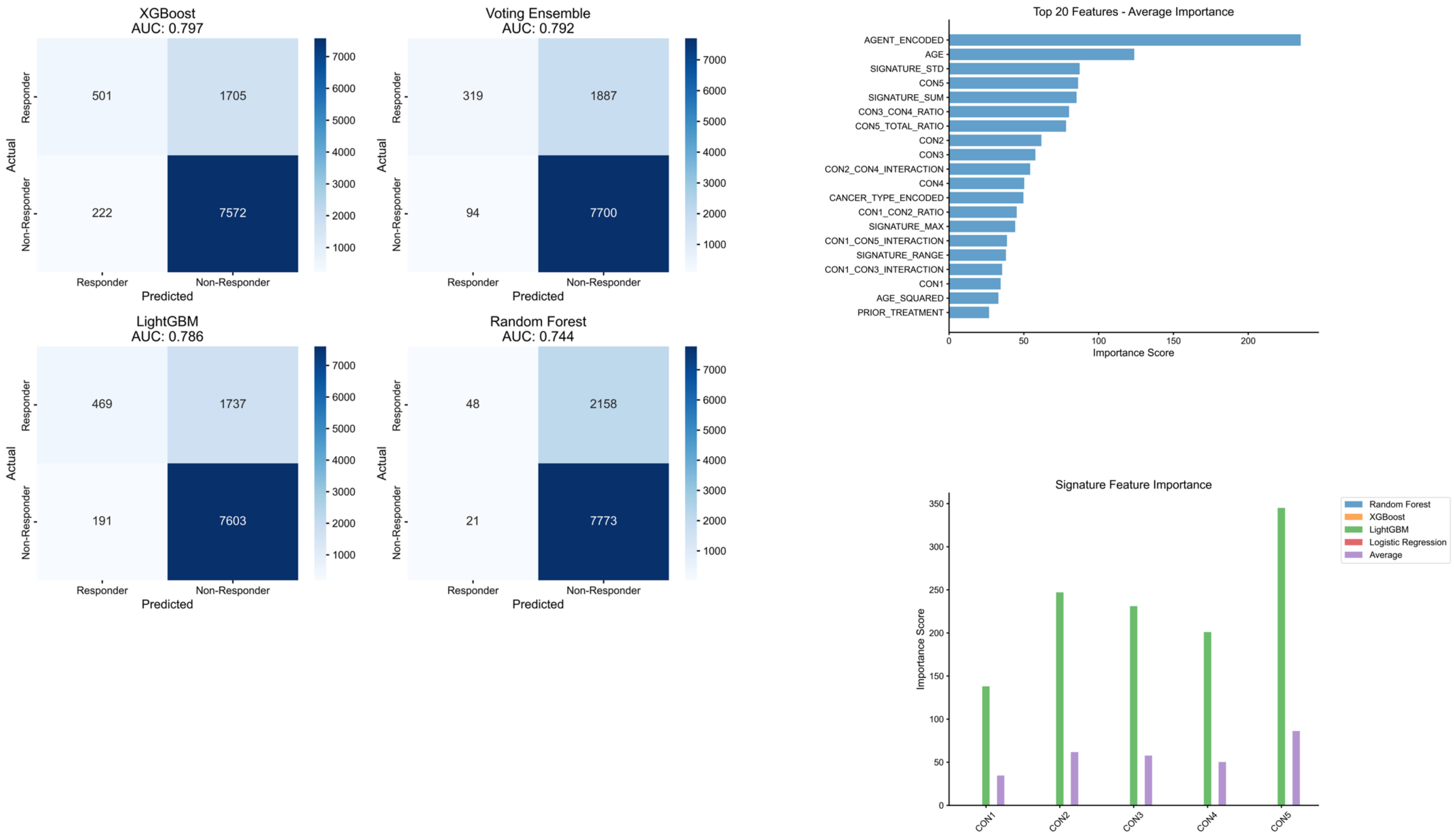

Supplementary Figure S9. Predictive models validation set scores and feature importance

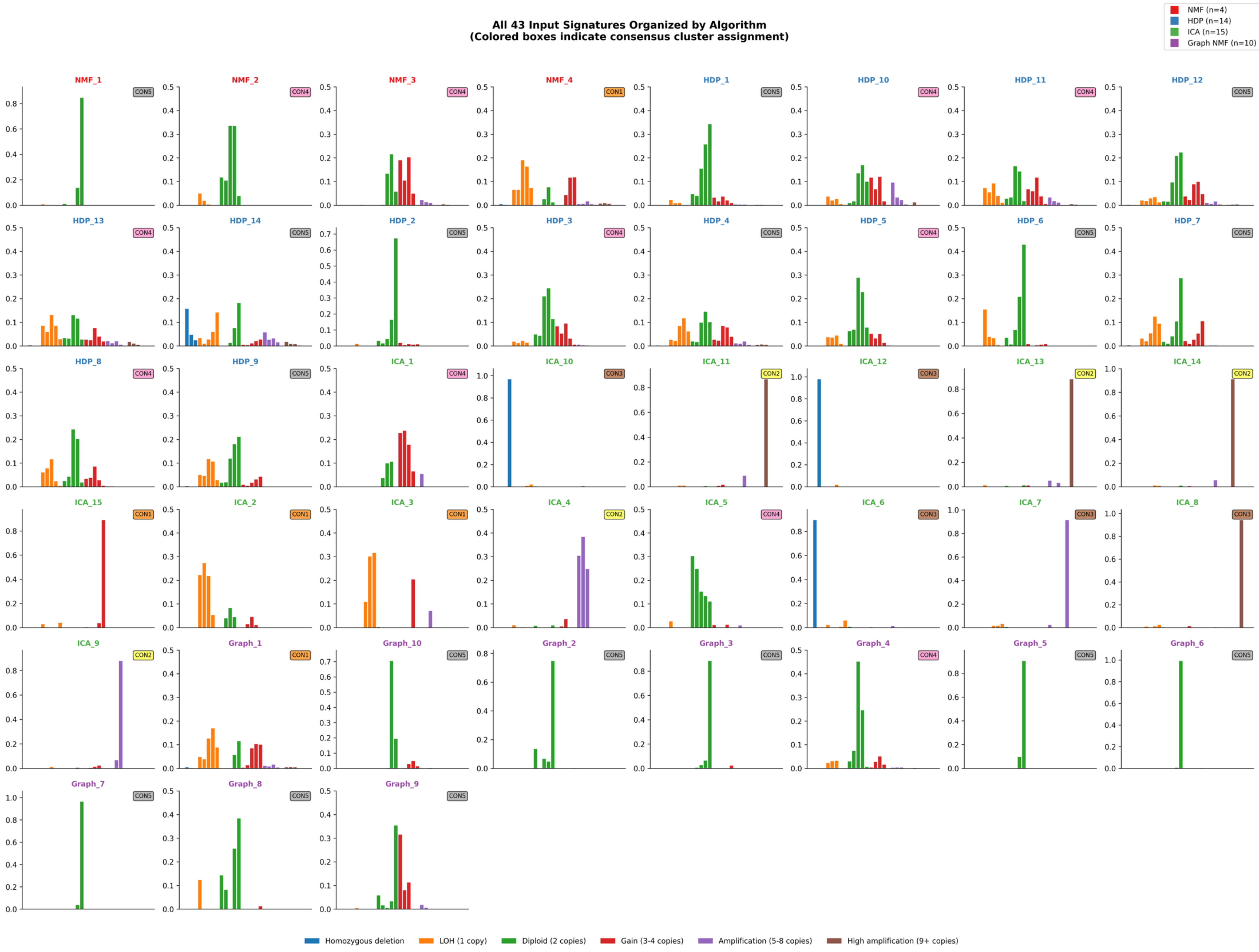

Supplementary Figure S10. All 43 input signatures, organized by algorithm.
